# A network pharmacology-based approach to explore potential targets of *Caesalpinia pulcherima*: an updated prototype in drug discovery

**DOI:** 10.1038/s41598-020-74251-1

**Published:** 2020-10-14

**Authors:** Nikhil S. Sakle, Shweta A. More, Santosh N. Mokale

**Affiliations:** Y. B. Chavan College of Pharmacy, Dr. Rafiq Zakaria Campus, Aurangabad, Maharashtra 431001 India

**Keywords:** Breast cancer, Target identification, Target validation

## Abstract

*Caesalpinia pulcherima* (CP) is a traditional herb used for the treatment of asthma, bronchitis, cancer, anti-bacterial, anti-fungal and as abortifacient. In the present study, bioactive components and potential targets in the treatment of breast cancer validated through in silico, in vitro and in vivo approach. The results for the analysis were as among 29 components, only four components were found active for further study which proved the use of CP as a multi-target herb for betterment of clinical uses. The results found by PPI states that our network has significant interactions which include the ESR-1, ESR-2, ESRRA, MET, VEGF, FGF, PI3K, PDK-1, MAPK, PLK-1, NEK-2, and GRK. Compound-target network involves 4 active compound and 150 target genes which elucidate the mechanisms of drug action in breast cancer treatment. Furthermore, on the basis of the above results the important proteins were fetched for the docking study which helps in predicting the possible interaction between components and targets. The results of the western blotting showed that CP regulates ER and EGFR expression in MCF-7 cell. In addition to this animal experimentation showed that CP significantly improved immunohistological status in MNU induced carcinoma rats. Network pharmacology approach not only helps us to confirm the study of the chosen target but also gave an idea of compound-target network as well as pathways associated to the CP for treating the complex metabolic condition as breast cancer and they importance for experimental verification.

## Introduction

Breast cancer (BC) is the malignant growth that begins in the breast cells. BC is the widespread cancer in India (in women) which accounts for 14% of all the cancers in women^1,2^. In general, 1 in 28 women are prone to have BC during her life span. The frequency rates in India begin to rise in the early thirties and peak at the age of 50–64 years^3^. In urban areas, 1 in 22 women are likely to have BC during her life span as compared to rural areas where 1 in 60 women develop BC in her life span^4^. Duration of survival of cancer patients is an important sign for knowing the result of treatment in any study. Since the 1990s, due to regular efforts in the diagnosis and treatment, the overall survival time of patients has been enhanced^5^. In cancer multiple genes participate which gradually alters the normal healthy cells into cancerous cells. Cancers have the capacity to develop resistance to conventional chemotherapy^6^. Thus, it is essential to recognize new therapeutic agents or promising targets. In current cancer therapy, new generations of drugs have targeted cancer specific proteins that are expressed in different cancers. Target specific cancer therapy minimizes the side effect profiles of conventional cytotoxic drugs^7^. In targeted therapy attempts are being made to design ligands with maximum selectivity to act on specific drug targets. Network pharmacology (NP) is an emerging discipline useful in drug discovery, which combines genomic technologies and system biology through computational biological tool. Network pharmacology, is an approach capable of describing complex relationships among biological systems, drugs and diseases^8^. It also clarifies the possible mechanisms of complex bio-actives through large data set analysis and determines the synergistic effects in cancer treatment^9^. Traditional Chinese medicine (TCM) is combination of complex herbal formulation and has been used for more than thousands of years in the treatment of different diseases and disorders for the prolongation of life expectancy in different parts of the world^10^. The network-target-based network pharmacology is a promising approach for the next-generation mode of drug research and development for TCM herbs or herbal formulae. It provides a new logical guide and technical route for developing and understanding TCM drugs' mechanisms of action. NP encourages the discovery of successful molecules, recognizing their interrelationship, elucidating the relationship between TCM formulae and diseases or TCM syndrome, establishing logical TCM drugs, as well as directing integrated use of TCM and conventional drugs^11–13^. CP decoction or infusion traditionally used as purgative, tonic in the treatment of convulsions, intermittent fevers, lung and skin diseases, cure bad cough, breathing difficulty and chest pain, treats inflammations, earache, muscular and rheumatic pain and various cardiovascular disease, cancer and other chronic diseases^14,15^.

In our previous study we have tried to investigate the effect of ethyl acetate fraction of CP in breast cancer rats and possible molecular mechanism involved by the CP^16^. Therefore, this study extends to use NP approach to establish the effect of CP in BC and to predict core targets and biological functions, pathways and mechanism of action. Molecular docking was also performed on selected active components and the key targets to validate the NP results.

## Results

### Components in CP

According to literature and TCMID, TCM, TCMSP databases 61 active components were found in CP. The selected components were further subjected to evaluate pharmacokinetics parameters i.e. ADME criteria for OB ≥ 30% and DL ≥ 0.18 values are shown in Table 1.

**Table 1:** The specific information of anti-breast cancer components in *Caesalpinia Pulcherima.*

MOL ID	Components	OB%	DL%	BBB
MOL000513	Gallic acid	31.69	0.04	− 0.54
MOL000089	Catechin	54.83	0.24	− 0.73
MOL000415	Rutin	3.2	0.68	− 2.75
MOL001002	Ellagic acid	43.06	0.43	− 1.41
MOL000098	Quercetin	46.43	0.28	− 0.77
MOL001111	α-Pinene	46.25	0.05	2.18
MOL001111	β-Pinene	44.77	0.05	2.29
MOL000023	Limonene	39.84	0.02	2.12
MOL001115	E-Verbenol	50.68	0.06	1.48
MOL000232	α-Terpineol	48.8	0.03	1.72
MOL007510	α-Copaene	37.81	0.08	2.04
MOL00036	E-Caryophyllene	29.7	0.09	2.07
MOL001177	β-Copaene	29.47	0.12	2.04
MOL001180	α-Muurolene	21.53	0.08	2.05
MOL001123	β-Muurolene	19.5	0.08	2.16
MOL004724	γ-Cadinene	21.34	0.08	2.09
MOL012609	E-Nerolidol	29.61	0.06	1.44
MOL013232	1-Epi-Cubenol	58.49	0.09	1.49
MOL001123	α-Phellandrene	19.5	0.08	2.16
MOL000911	α-Terpinene	33.95	0.02	2.1
MOL000257	β-Phellandrene	56.28	0.12	2.06
MOL000122	1,8-Cineole	43.75	0.05	2.27
MOL000202	γ-Terpinene	33.02	0.02	2.05
MOL000920	Linalool	49.37	0.04	0.74
MOL012618	Trans-linalool oxide (pyranoid)	22.91	0.07	1.07
MOL000608	Terpinen-4-ol	32.16	0.03	1.52
MOL000036	β-Caryophyllene	29.7	0.09	2.07
MOL001193	Caryophyllene oxide	32.67	0.13	1.76
MOL008549	Cyanidin 3-glucoside	58.99	0.24	− 0.04

### Screening of components of CP for breast cancer

Among the 61 components, only 29 components were found to relate with breast cancer as shown in Table 1. Using TCMSP the 29 components were subjected to OB and DL criteria filtering out with only four active components of CP based on the ADME criteria. The genes of each active component were fetched from Swiss target prediction whereas genes of the disease i.e., breast cancer were retrieved from GeneCards Human database.

### Construction and analysis of target PPI network

**Figure 1:**
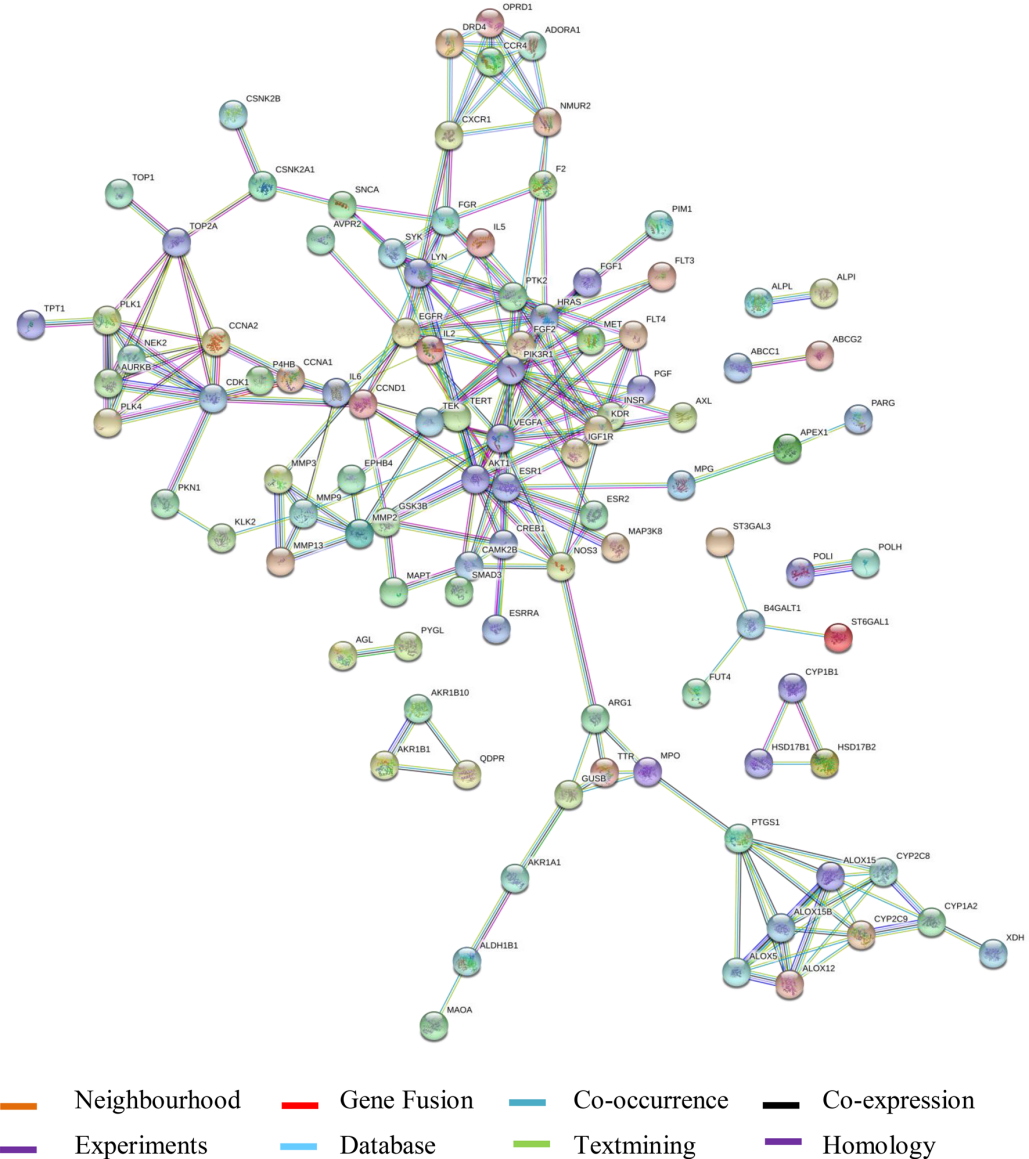
Protein–protein interaction network of CP in breast cancer (BC) targets obtained from STRING v_11.0 database (contributing co-author: Shweta A. More).

In order to enhance the visualization and understand the mechanism of the targets, it is important to study the PPI of the target genes. The target genes of the corresponding components were subjected to STRING v_11 to visualize and construct the PPI network for the same. The high-confidence target protein interaction data was set with a score level greater than 0.9. The interactions between the target proteins is depicted in Fig. 1, which comprises of total 124 nodes, 233 edges; each edge represents PPIs. The other parameter is average node degree which values at 3.76 and local clustering co-efficient: 0.538 corresponds to the number of targets that are connected to the network. Degree plays an important role in showing the role of proteins interaction and nodes of network. The PPI network shows the targets involved in breast cancer are ESR-1, ESR-2, ESRRA, MET, VEGF, FGF, PI3K, PDK-1, MAPK, PLK-1, NEK-2, and GRK which are the major targets in breast cancer. Along with ESR-1; EGFR, MET, and VEGFR are located centrally in the network which indicates the role of proteins in the pathogenesis of breast cancer. Basically, ESR pathway is a forthcoming starting point to discover the mechanisms of breast cancer. The main factor in ESR pathway is estrogen which is found to be an integral component involved in the development and maturation of breast. The involvement of estrogen receptors are also seen in other pathological processes including breast cancer, endometrial cancer, and osteoporosis. So, PPI network and pathway analysis of novel genes were carried out for the recognition of critical genes related to the breast cancer.

### Arrangement and construction of disease-target network

**Figure 2:**
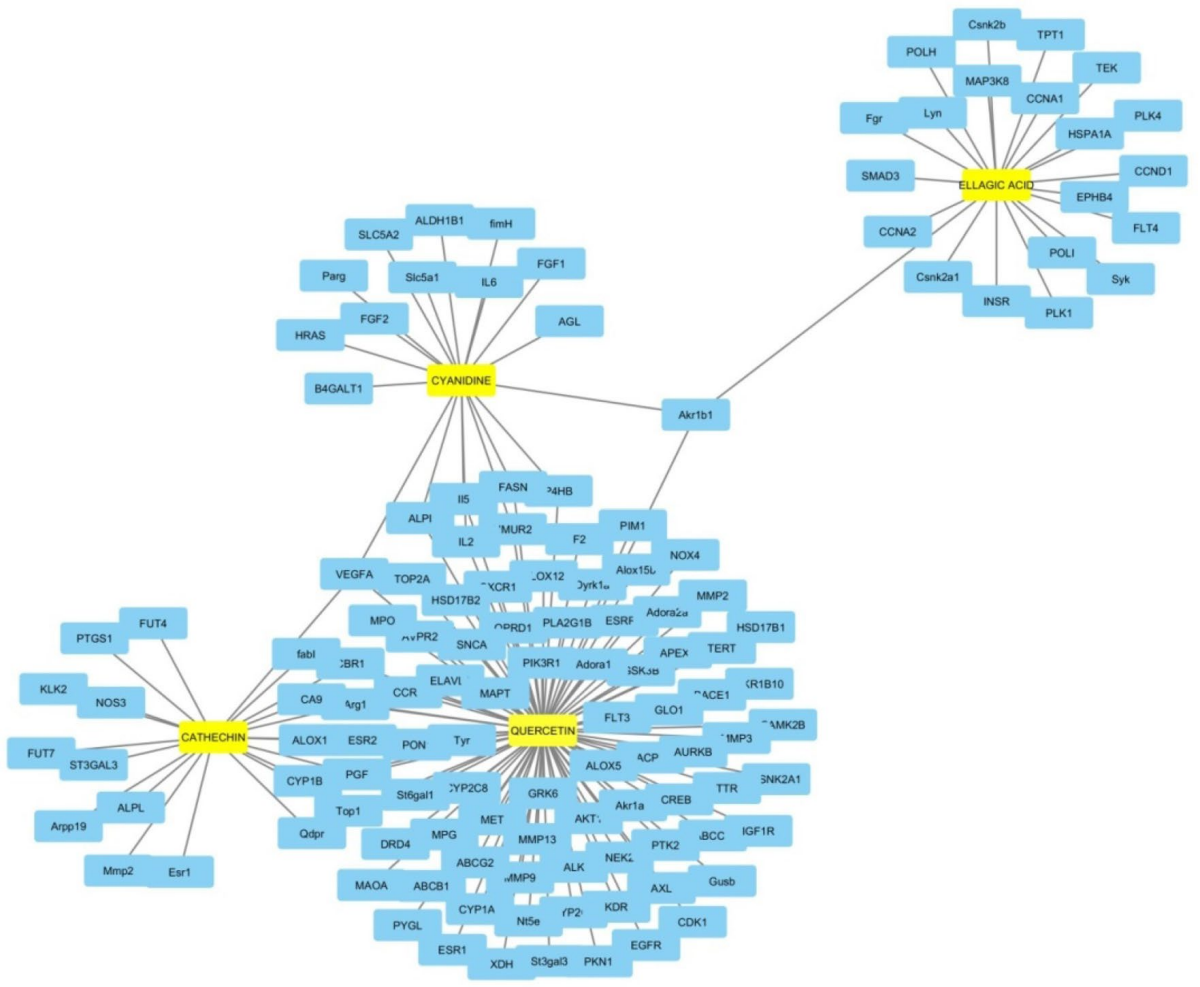
Compound-target-breast cancer network constructed by Cytoscape v_3.7.1 (contributing co-author: Shweta A. More).

To study the signaling pathway and function of the selected target genes, the data was imported to Cytoscape to construct compound-target network. In Fig. 2, the compound-target-disease interaction network was constructed which elucidate the mechanisms of drug action in the breast cancer treatment. It consists of 4 ingredients, and 150 interactive target proteins. In this network, we found that many targets were hit by multiple components. This fact inferred that the active biochemical of CP might influence these targets synergistically; it has therapeutic effects on other disease and disorders additionally to BC. The details of three topological parameters i.e. Betweenness Centrality, Closeness Centrality, and Degree are given in Table 2 which gives an important role of each target in the network structure.

**Table 2:** Important nodes with network analyzer results.

Name	Betweenness centrality	Closeness centrality	Clustering coefficient	Degree	Topological coefficient
AKT1	0.15105568	0.42124542	0.23913043	24	0.16908213
GSK3B	0.00818	0.3422619	0.33333333	7	0.28011204
CDK1	0.04618775	0.3133515	0.42222222	10	0.25
CCNA2	0.05253746	0.32951289	0.48888889	10	0.23658537
CSNK2B	0	0.22157996	1	2	0.59090909
CSNK2A1	0.01619132	0.24364407	0.16666667	4	0.27777778
VEGFA	0.05497117	0.40636042	0.31481481	28	0.18434874
KDR	0.00489856	0.33923304	0.55555556	10	0.34782609
NOS3	0.03367264	0.38333333	0.36363636	12	0.23870056
AURKB	0	0.26436782	1	6	0.49019608
PLK1	0.04334413	0.30913978	0.47222222	9	0.2251462
HRAS	0.09982428	0.40069686	0.25846154	26	0.18131868
EGFR	0.10619747	0.40636042	0.27272727	23	0.1970547
TOP2A	0.03747492	0.26995305	0.44444444	9	0.32716049
TOP1	0	0.21296296	0	1	0
IL5	0.00137232	0.35060976	0.73333333	6	0.38461538
IL2	0.00626375	0.36741214	0.58333333	9	0.3132969
CCNA1	0.00101686	0.3042328	0.7	5	0.39230769
PIK3R1	0.1135341	0.4020979	0.24074074	28	0.17176871
INSR	1.72E−04	0.3314121	0.9	5	0.47916667
IGF1R	0.0013309	0.33625731	0.58333333	9	0.38164251
NEK2	0	0.26436782	1	6	0.49019608
CREB1	0.05089584	0.37337662	0.31818182	12	0.2295082
FLT4	1.24E−04	0.32122905	0.80952381	7	0.42857143
AGL	0	1	0	1	0
PYGL	0	1	0	1	0
ESR1	0.05830291	0.39383562	0.36263736	14	0.21978022
CCND1	0.10797187	0.41071429	0.33333333	18	0.20422535
MMP9	0.08713433	0.39655172	0.375	16	0.22420635
PLK4	0	0.26436782	1	6	0.49019608
MAPT	0.00399207	0.30831099	0.3	5	0.30344828
TERT	0.02200057	0.33527697	0.52380952	7	0.35428571
PGF	0.0063316	0.35276074	0.5	12	0.29716981
TPT1	0	0.23662551	0	1	0
FGF1	0.00111343	0.33823529	0.63636364	11	0.34042553
FGF2	0.07449146	0.39383562	0.37662338	22	0.21306818
MMP2	0.00467167	0.35714286	0.57777778	10	0.29423077
MET	6.53E−04	0.34124629	0.77777778	9	0.4
SMAD3	0.00199754	0.33625731	0.46666667	6	0.34848485
ALOX5	6.10E−05	0.21821632	0.9047619	7	0.77777778
PTGS1	0.09914773	0.26932084	0.67857143	8	0.50961538
FLT3	0.00238706	0.33045977	0.66666667	7	0.41883117
TEK	3.95E−04	0.33045977	0.71428571	7	0.44480519
ESR2	0.11758808	0.37828947	0.47619048	7	0.30295567
LYN	0.03300857	0.36741214	0.45454545	11	0.24633431
SYK	0.00326702	0.33045977	0.61904762	7	0.35191638
HSD17B1	0	0.23760331	1	3	0.66666667
HSD17B2	0	0.23760331	1	3	0.66666667
B4GALT1	0.5	0.8	0.33333333	3	0.55555556
ST3GAL3	0	0.66666667	1	2	0.75
FGR	0.01597583	0.34638554	0.46428571	8	0.25510204
ST6GAL1	0	0.5	0	1	0
PTK2	0.0039192	0.34534535	0.51515152	12	0.30065359
MMP3	3.67E−04	0.33823529	0.86666667	6	0.37037037
MPG	0.02074822	0.29187817	0	2	0.5
APEX1	0.02021823	0.24678112	0	4	0.25
IL6	0.21357151	0.42279412	0.22134387	23	0.16205534
ALOX15	0.00360723	0.22373541	0.75	8	0.60227273
ALOX12	6.10E−05	0.21821632	0.9047619	7	0.77777778
ARG1	0.03662179	0.32951289	0.4	6	0.27222222
POLI	0.0173913	0.22373541	0	2	0.5
POLH	0	0.18312102	0	1	0
MAP3K8	0	0.29715762	0	1	0
CYP1B1	0.11543811	0.30263158	0.3	5	0.28571429
GUSB	0.04549909	0.27058824	0.5	4	0.45
MPO	0.13589779	0.33823529	0.4	6	0.27027027
ALOX15B	0	0.21780303	1	6	0.83333333
AXL	0	0.29262087	1	2	0.65517241
ABCC1	0	0.23046092	0	1	0
ABCG2	0.0173913	0.2987013	0	2	0.5
ESRRA	0	0.28822055	1	2	0.72222222
SNCA	0.02432927	0.29113924	0.23809524	7	0.28571429
CXCR1	0.0825549	0.33527697	0.30555556	9	0.21604938
CAMK2B	0.00167032	0.32122905	0.16666667	4	0.35810811
F2	0.00591872	0.35493827	0.3	5	0.35686275
ALDH1B1	0.01815372	0.20282187	0.16666667	4	0.42857143
AKR1A1	0.03250421	0.22373541	0	3	0.38888889
CYP2C9	0.00360723	0.22373541	0.75	8	0.60227273
CYP1A2	0.03517144	0.24416136	0.3	5	0.45
AKR1B1	0.00833383	0.18341308	0.2	5	0.4
QDPR	0.02290496	0.20390071	0.33333333	3	0.45833333
AVPR2	0.0173913	0.29113924	0	2	0.5
CYP2C8	0.00280828	0.22330097	0.80952381	7	0.63636364
PIM1	0	0.28822055	1	2	0.61111111
EPHB4	3.05E−04	0.32303371	0.33333333	3	0.47747748
OPRD1	0	0.27251185	1	5	0.50769231
DRD4	0.0465845	0.27777778	0.66666667	6	0.4047619
PARG	0	0.19827586	0	1	0
P4HB	0	0.29792746	0	1	0
MAOA	0.03380981	0.22637795	0	3	0.375
ADORA1	0.00744264	0.27380952	0.66666667	6	0.42307692
TTR	0.00942748	0.31944444	0.83333333	4	0.36111111
MMP13	4.88E−05	0.32485876	0.93333333	6	0.42682927
KLK2	0.00705145	0.29262087	0	2	0.5
FUT4	0.5	0.8	0.33333333	3	0.55555556
PKN1	0.00167811	0.25054466	0	2	0.5
NMUR2	0.04100187	0.32303371	0.52380952	7	0.2406015
AKR1B10	0.00183066	0.18282989	0.66666667	3	0.5
HSD17B8	0.03606286	0.24159664	0.5	4	0.46428571
POLD3	0.00607311	0.25842697	0	2	0.5
XDH	0	0.1965812	0	1	0
CCR4	0	0.27251185	1	5	0.50769231
ALPL	0	1	0	1	0
ALPI	0	1	0	1	0
NEU1	0	0.24838013	0	1	0
IAPP	0	0.2259332	0	1	0
DYRK1A	0.001214	0.28255528	0.33333333	3	0.45098039
ELAVL1	0.00120149	0.31944444	0.66666667	4	0.39102564
ALK	0	0.31081081	1	2	0.77142857
PLA2G1B	0	0.2173913	1	5	0.84444444
GLO1	0.00350393	0.19166667	0	2	0.6
ARPP19	0	0.23908524	0	1	0
FUT7	0	0.5	0	1	0
NOX4	7.19E−05	0.30503979	0.66666667	3	0.51851852
BACE1	0.0344775	0.28606965	0	2	0.5
GRK6	0	0.25164114	0	1	0
CBR1	0.0027995	0.18821604	0	2	0.6
FASN	0	0.29715762	0	1	0
ADORA2A	0.01137633	0.2804878	0	2	0.5
NT5E	0.00173277	0.23279352	0	2	0.5
ABCB1	0	0.28967254	0	1	0
CA9	0	0.30585106	1	2	0.70833333
SLC5A1	0	0.28967254	0	1	0
HSPA1A	0	0.29792746	0	1	0
PON1	0	0.29792746	0	1	0

### GO gene enrichment analysis and KEGG pathway annotation

**Figure 3:**
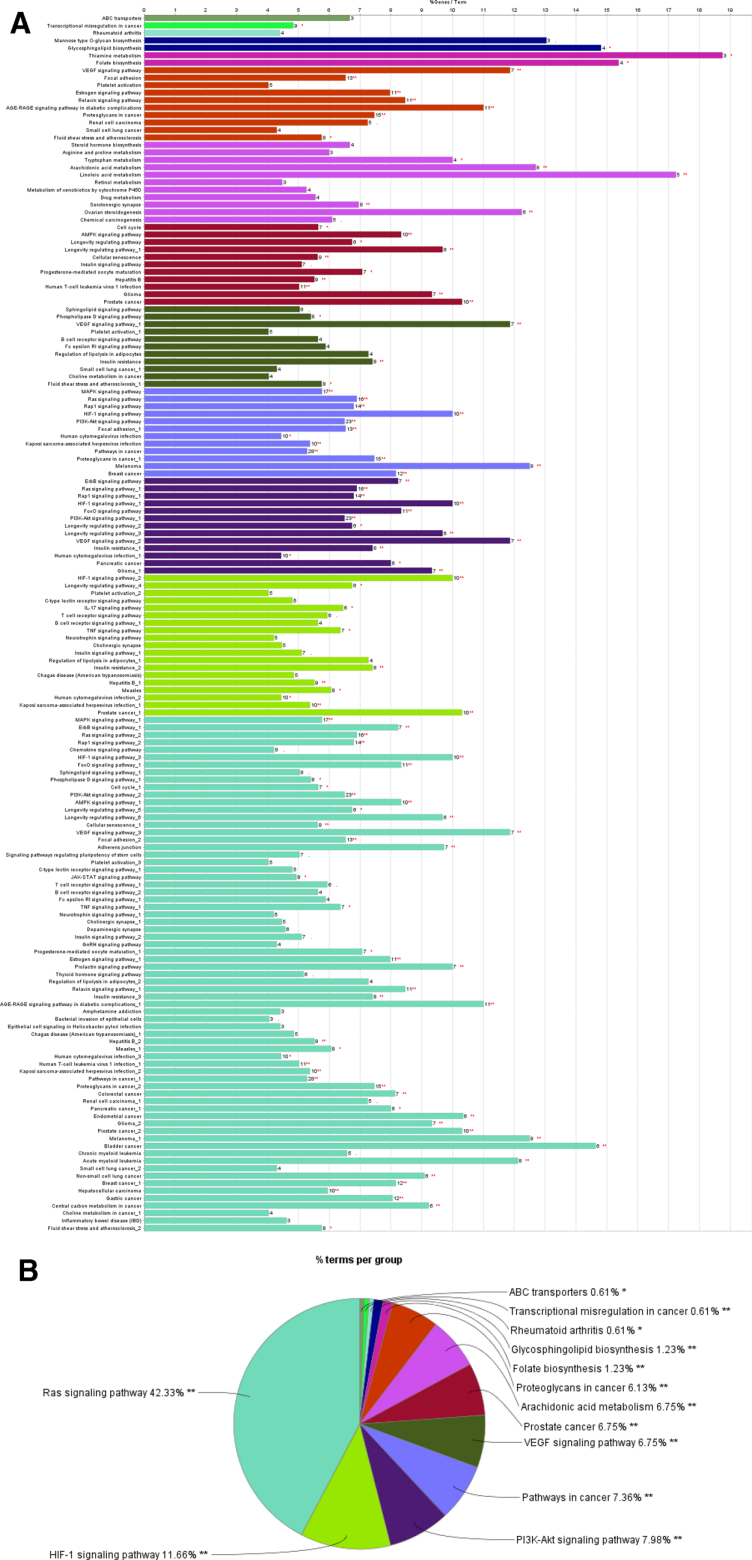
(**A**) Kyoto encyclopaedia of genes and genomes pathway (Contributing co-author: Shweta A. More). (**B**) Gene ontology enrichment analysis (contributing co-author: Shweta A. More).

GO enrichment analysis was carried out to analyse the target proteins. The setting for the ClueGO was set for three criteria to analyse the target genes for GO biological (Table 3), Go molecular (Table 4), and GO cellular (Table 5) and the most important parameter as KEGG pathway (Fig. 3A). The GO term fusion was restricted to pV ≤ 0.005 that is based on the false discovery rate (Benjamini-Hochberg). The Ras-Raf-MAPK signaling pathway is a key route for the ErbB family, as is the PI3K/AKT pathway, both of which results in alteration of cell proliferation and apoptosis. As far as breast cancer is considered, over-expression of ErbB receptor may lead to Ras activation. So to analyse the statement, GO and KEGG analysis was carried to determine the signaling pathway and following pathways were found to associate: PI3K-Akt, MAPK, ErbB, Ras, Chemokine, HIF-1, FoxO, sphingolipid, AMPK, VEGF, JAK-STAT, TBF, insulin, GnRH, estrogen signaling pathway, prolactin signaling pathway, thyroid hormone signaling pathway, and relaxin signaling pathway (Fig. 3B).

**Table 3:** GO biological process.

Description	Count in gene set	False discovery rate
Organic substance metabolic process	112	4.42 × 10^–21^
Response to organic substance	67	4.42 × 10^–21^
Metabolic process	113	1.65 × 10^–20^
Cellular response to chemical stimulus	64	1.90 × 10^–20^
Phosphate-containing compound metabolic process	57	2.01 × 10^–20^
Cellular metabolic process	108	1.09 × 10^–19^
Protein phosphorylation	40	1.09 × 10^–19^
Response to chemical	76	3.80 × 10^–19^
Regulation of cell death	49	1.84e × 10^–19^
Organonitrogen compound metabolic process	84	2.00 × 10^–18^

**Table 4:** GO molecular function.

Description	Count in gene set	False discovery rate
Catalytic activity	100	4.23 × 10^–30^
Protein kinase activity	35	3.07 × 10^–20^
Phosphotransferase activity, alcohol group as acceptor	37	7.44 × 10^–20^
Transferase activity, transferring phosphorus-containing groups	40	2.82 × 10^–19^
Kinase activity	37	7.10 × 10^–19^
Ion binding	86	3.07 × 10^–16^
Protein tyrosine kinase activity	19	2.31 × 10^–15^
Anion binding	55	1.68 × 10^–14^
Drug binding	42	5.65 × 10^–13^
Transferase activity	48	5.69 × 10^–13^

**Table 5:** GO cellular component.

Description	Count in gene set	False discovery rate
Extracellular region	45	1.29 × 10^–08^
Cytoplasmic part	93	1.71 × 10^–07^
Cell periphery	65	2.31 × 10^–07^
Plasma membrane	63	6.99 × 10^–07^
Cytoplasm	100	3.59 × 10^–06^
Plasma membrane part	39	1.79 × 10^–05^
Membrane	81	3.08 × 10^–05^
Intracellular organelle lumen	58	5.02 × 10^–05^
Cytosol	56	5.64 × 10^–05^
Cell part	120	6.81 × 10^–05^

CP can be used for the treatment of other conditions also as hepatitis B, measles, human T-cell leukemia virus 1 infection, Kaposi sarcoma-associated herpes virus infection. As the constituents of CP was selected to examine the effects on breast cancer but via KEGG analysis (Table 6) it was found responsible in many cancers as colorectal cancer, renal cell carcinoma, pancreatic cancer, endometrial cancer, prostate cancer, melanoma, bladder cancer, small cell lung cancer, non-small cell lung cancer, hepatocellular carcinoma, and gastric cancer. Owing to the facts and visualization CP may be used as novel drug for the treatment of various diseases and disorder.

**Table 6:** KEGG pathways.

Description	Count in gene set	False discovery rate
Pathways in cancer	28	1.08 × 10^–15^
PI3K-Akt signaling pathway	24	1.11 × 10^–15^
EGFR tyrosine kinase inhibitor resistance	12	3.19 × 10^–11^
Metabolic pathways	33	1.13 × 10^–10^
Ras signaling pathway	16	1.46 × 10^–10^
Proteoglycans in cancer	15	1.78 × 10^–10^
MAPK signaling pathway	17	3.66 × 10^–10^
Endocrine resistance	11	1.93 × 10^–09^
AGE-RAGE signaling pathway in diabetic complications	11	2.33 × 10^–09^
Rap1 signaling pathway	14	2.33 × 10^–09^

### Molecular modelling: docking of the active components

**Figure 4:**
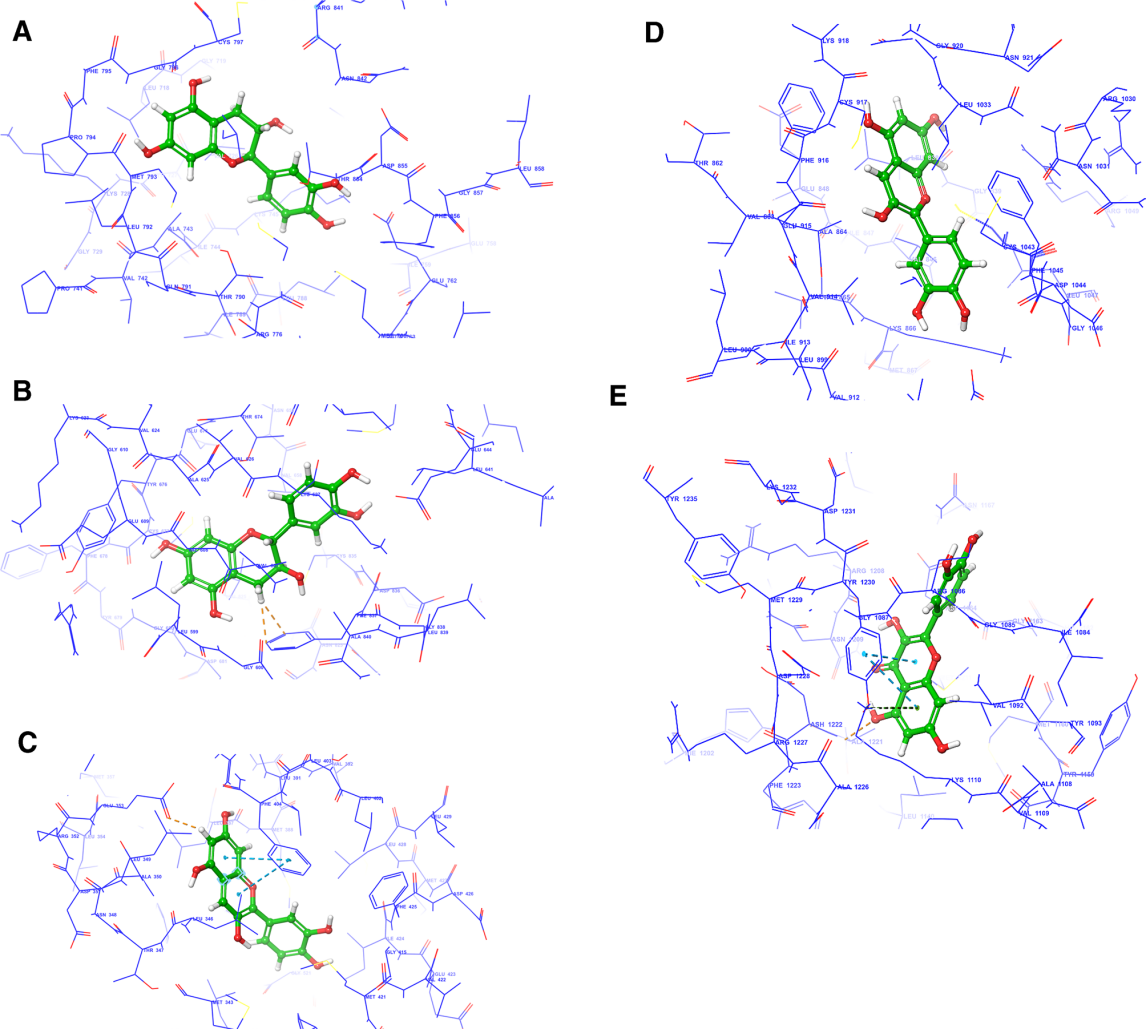
Docking analysis of components and targets. (**A**) Catechin and EGFR (GLU 762). (**B**) Catechin and PDGFR (PHE 837). (**C**) Cyanidin and 1ERR (LEU 346). (**D**) Cyanidin and VEGFR (LEU 838). (**E**) Quercetin and c-Met (ALA 1221) (contributing author: Santosh N Mokale).

After analysing the pathways and diseases and disorder related to the genes, it is important to study structure based design of the component, as well as its ability to predict the binding-conformation of small molecule ligands to the appropriate target binding site. The main reason behind selecting the proteins is that they played a major role in PPI, Compound-target network and in KEGG analysis; as well as these proteins are found to play a vital role in the mechanism of BC. Table 7 and Fig. 4A–E gives the detail about docking score and poses of active components i.e., cyanidin, catechin, ellagic acid, and quercetin against c-MET (PDB Id: 5EYD), EGFR (PDB Id: 4ZAU), PDGFR-α/β (PDB Id: 5GRN), VEGFR (PDB Id: 2OH4), and ERR (PDB Id: 1ERR-α).

**Table 7:** Docking score of tyrosine kinase (interacting amino acid).

Active constituents	Docking Score of tyrosine kinase (interacting amino acid)				
	c-Met (PDB ID: 5EYD)	EGFR (PDB ID: 4ZAU)	PDGFR α/β (PDB ID: 5GRN)	VEGFR family (PDB ID:2OH4)	ERR (PDB ID: 1ERR-α)
Catechin	− 6.6651 (TYR 1230)	− **7.969 (GLU 762)**	− **10.392 (PHE 837)**	− 7.793 (CYS 917)	− 9.533 (MET 421)
Cyaniding	− 7.315 (ARG 1208)	− 7.747 (GLU 762)	− 10.302 (GLU 644)	− **11.233 (LEU 838)**	− **9.895 (LEU 346)**
Ellagic acid	− 7.348 (GLY 1163)	− 5.792 (MET 793)	− 4.396 (VAL 815)	− 4.727 (ARG 1025)	− 8.197 (LEU 346)
Quercetin	− **7.576 (ALA 1221)**	− 7.417 (GLU 762)	− 10.056 (LEU 599)	− 10.224 (CYS 917)	− 9.378 (GLU 353)

Bold signifies that among each class of tyrosine kinase the maximum score of the active component.

### In vitro experimental validation

**Figure 5:**
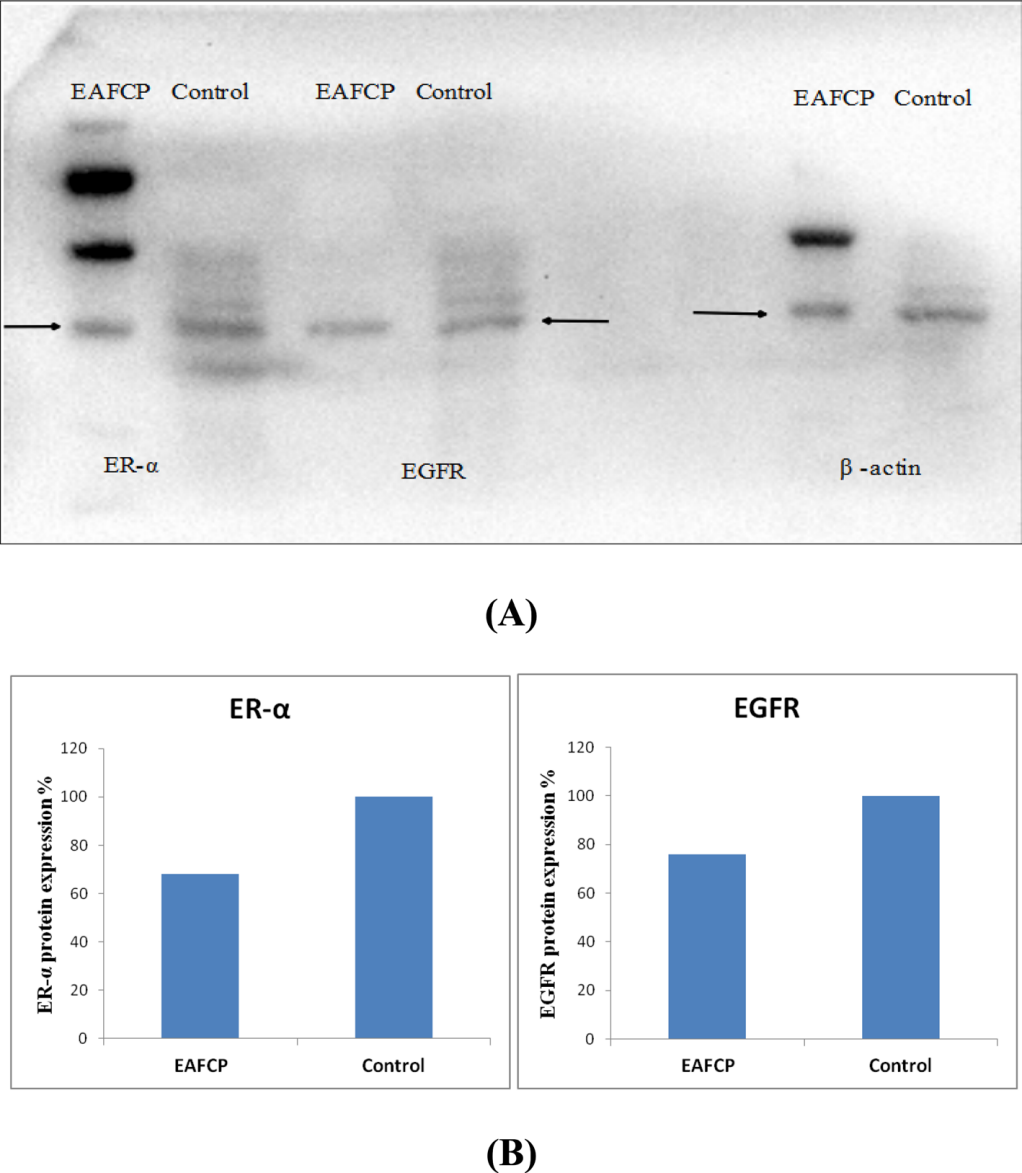
Influence of EAFCP treatment on expression ER signaling proteins in MCF-7 cell. (**A**) Representative western blot images of ER-α and EGFR protein expression. β-actin was used as a control. (**B**) Relative protein concentration of ER- α and EGFR respect to β-Actin (contributing co-author: Nikhil S. Sakle).

The KEGG analysis indicated that PI3K-Akt, Ras-Raf-MAPK and estrogen signaling pathway are a key route associated with breast cancer treatment by CP. To verify the reliability of obtained targets in network pharmacology analysis, we have conducted western blot analysis for ER and EGFR protein expression to confirm signaling pathway in breast cancer effects of the CP. The expression of ER and EGFR were declined after 24 h in MCF-7 cells treated with EAFCP at dose level 200 µg/ml. The obtained results indicate that EAFCP may suppress estrogen regulated EGFR mediated signaling pathway (Fig. 5).

### In vivo experimental validation

**Figure 6:**
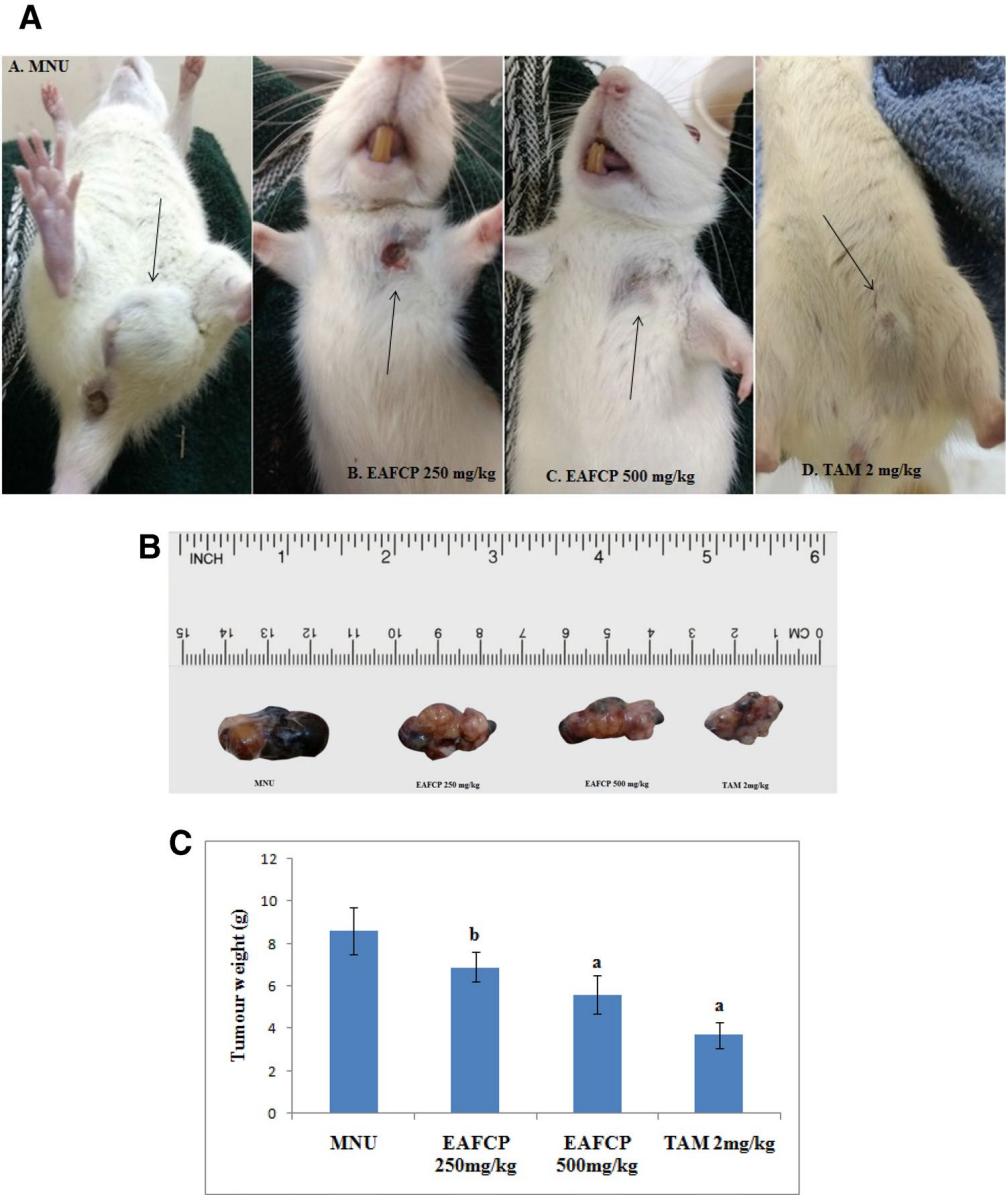
In vivo experimental study. (**A**) Representative rats in MNU control and treated group. (**B**) Representative images of tumours obtained from MNU control and treated rats. (**C**) Weight of tumours (g) (mean ± SEM; ^a^*p* < 0.001, ^b^*p* < 0.01) (contributing co-author: Nikhil S. Sakle).

**Figure 7:**
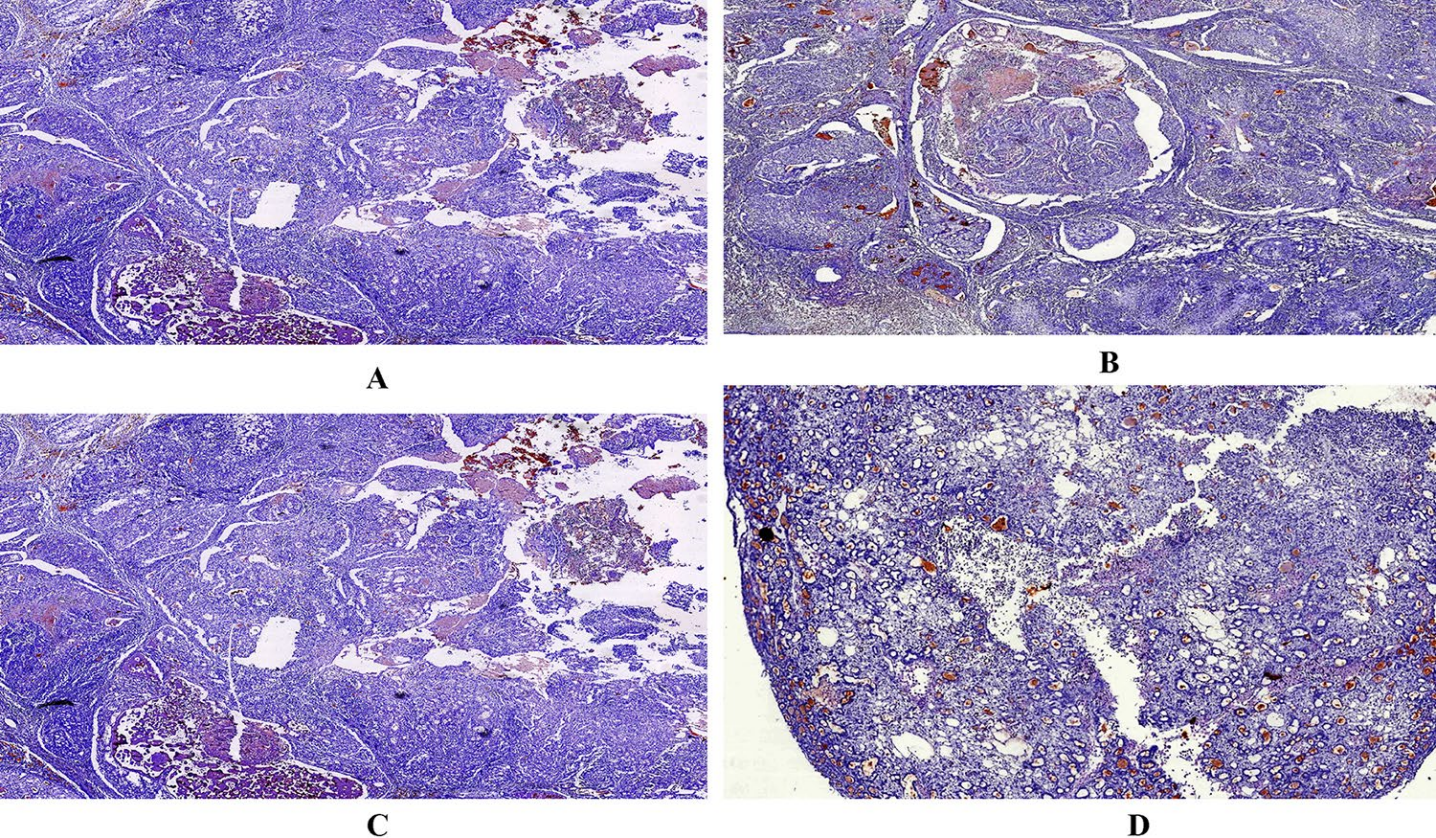
Significant ER-α immunohistological changes in mammary gland of control and treated rats after 04 week of treatment. All the groups receive i.p. dose of MNU at 50 mg/kg, body weight. (**A**) MNU Control. (**B**) EAFCP 250 mg/kg. (**C**) EAFCP 500 mg/kg. (**D**) TAM 2 mg/kg (contributing co-author: Nikhil S. Sakle).

In this part to reveal the molecular mechanism of EAFCP (ethyl acetate fraction of *Caesalpinia pulcherima*) in treating MNU (N-Methyl-N-nitrosourea) induced mammary carcinoma, the anti-tumour effect improved after treatment for 30 days. EAFCP 500 mg/kg and TAM (Tamoxifen) 2 mg/kg tumour-bearing rats were significantly more resistant to the development tumour than control rats and observed decreased tumour development (Fig. 6A–C). Consequently, we have examined the effect of treatment on the density of ER-α expression by immunohistochemistry (Fig. 7A–D). After EAFCP and TAM treatment, ER-α immunoreactivity inside the nucleus was reduced significantly. The results strongly established that the treatment worsen tumour development by interfering ER.

## Discussion

As far as traditional approach is considered “one drug, one target” theory of drug design is used, in contradictory network pharmacology which aims to explore the correlation of drugs and diseases, based on the multi-targeted therapy^17^. Novelty of this approach includes the use of systems biology, network analysis, connectivity, and redundancy. NP studies was successfully used to identify the novel targets and to determine the unknown signaling pathways interact with compounds^18,19^. The NP approach provides new insights into the systemic connection between therapeutic targets, and a disease as a whole and provides a powerful and promising tool for the clarification of disease mechanisms at a systemic level and the discovery of potential bioactive ingredients^20^. In this context the present study generated a novel network which gives a general view of molecular mechanism of CP.

Active components found in CP are ellagic acid, gallic acid, cyanidine, catechin, quercetin, rutin, β-sitosterol, myricetin, flavonoids, homo-flavonoids, pulcherrimin, lupeol, glycosides and phenols. The components were screened for its DL and OB criteria and thus, four active components i.e. quercetin, ellagic acid, cyanidine, and catechin were found suitable for further studies. BC network constructed through the plant bioactive target followed by recognition of targets associated with BC pathway. The network reveals the potential of 4 CP bioactives to modulate the BC by the interactions of 150 proteins through multiple pathways. These components inhibit cells proliferation, induces cell cycle arrest and apoptosis in different cancer cell types^21–25^. Through PPI interaction of the genes we found 124 nodes and 233 edges. ESR-1, ESR-2, ESRR-A, MET, FGF, VEGF, PI3K, PDK-1, MAPK, PLK-1, NEK-2, and GRK were likely to be key genes in the development of BC. GO and KEGG analysis revealed several pathways as well as other disease and disorders for the selected genes. The GO enrichment analysis showed the direct involvement of bioactive in the regulation of BC. KEGG pathway analysis proved that estrogen signaling pathway and ErbB signaling pathway may be crucial signaling pathway in the selected network which helps to support that CP may be used for BC treatment. Other than estrogen signaling pathway and ErbB, Ras, Chemokine, HIF-1, FoxO, sphingolipid, PI3K-Akt, AMPK, VEGF, JAK-STAT, TBF, insulin signaling pathway, GnRH, estrogen signaling pathway, prolactin signaling pathway, thyroid hormone signaling pathway, and relaxin signaling pathway were also found, suggesting the use of CP in multi-targets. Adding on the evidence for CP it can be used in colorectal cancer, renal cell carcinoma, pancreatic cancer, endometrial cancer, prostate cancer, melanoma, bladder cancer, small cell lung cancer, non-small cell lung cancer, hepatocellular carcinoma and gastric cancer. In addition to this, the docking study was carried for the validation of targets. It also screens the affinity between the components and targets, which can directly clarify their structure–activity relationship. On the basis of the results obtained in network pharmacology, therapeutic effect of CP was investigated by western blotting, signifying that EAFCP treatment could regulates ER signaling pathway and suggesting that breast cancer can be treated through a complex system with multi-component target disease interaction. Our earlier preclinical study on the phytochemicals from CP decrease cell proliferation and induce apoptosis, significantly improved the pathological conditions of MNU induced breast cancer rat tissue suggesting their involvement in BC treatment^16^. It is not yet known whether giving CP alone or with chemotherapeutic agent will enhances the activity in treating patients with breast cancer which is future scope of the present study.

## Conclusion

This study scientifically investigates the pharmacological mechanism of CP in the treatment of breast cancer through network pharmacology, docking analysis, western blotting and in vivo animal study. It is in addition worth mentioning that network pharmacology has great advantages in clearing up the mechanism of CP as TCM.

## Materials and methods

The following parameters are important in order to construct a network: (1) identification and confirmation of compounds using chemical databases; (2) selection of compounds on the basis of pharmacokinetic parameter i.e. ADME (absorption, distribution, metabolism, and excretion) criteria; (3) selected compounds were further subjected to understand protein interaction and to obtain the relevant information by using publicly available database or tools; (4) genes related to target disease i.e., breast cancer were identified using human disease database and common genes of target and compounds were selected; (5) construction and analysis were carried out to understand the interaction and molecular mechanisms using visualization software; and (6) to perform docking study of the active actives.

### Chemical databases

The chemical components of CP were identified through literature and the Traditional Chinese Medicine Ingredient Database^26^ (TCMID, https://www.megabionet.org/tcmid/); the TCM Database@Taiwan^27^ (https://tcm.cmu.edu.tw/), most comprehensive databases on global scale. The chemical components were subjected to database called Traditional Chinese Medicine Systems Pharmacology^28^ (TCMSP, https://lsp.nwu.edu.cn/tcmsp.php) to screen for breast cancer. TCMSP helps to promote integration of both traditional as well as modern medicines in order to accelerate the drug discovery which builds the framework for system pharmacology and covers the ADME information. Chemical structures, synonyms, molecular weight, canonical SMILES and physicochemical properties were collected with the help of ChEMBL^29^ (https://www.ebi.ac.uk) and Pubchem^30^ (https://pubchem.ncbi.nlm.nih.gov).

### Evaluation of pharmacokinetics parameters

The selected components were further screened for oral bioavailability and drug-likeness pharmacokinetics parameters which include ADME. The ADME characteristics of the drug indicate the ratio of the oral drug to oral dosage of the blood circulatory system. The parameters to access the components druggability is analysed according to the set parameters as oral bioavailability (OB ≥ 30) value and drug-likeness (DL ≥ 0.18) indices recommended by TCMSP^28^. Among all the selected components, only the components which fit in the criteria were selected for construction of network.

### Identification of target genes and construction of target PPI (protein–protein interaction) network

PPI is important aspect to study the involvement of proteins in various biochemical processes in order to understand the cellular organization, bioprocess and functions. This can be done by using the virtual screening database called STRING 11.0 (https://string-db.org/)^31^. The genes of the selected components were uploaded to STRING to get the information about PPIs. The setting for generating the PPI network was in accordance with *‘Homo sapiens’* and the confidence in the interaction among the target protein was set to the highest confidence data > 0.9. The network nodes represent proteins whereas the edge represents associated protein–protein.

### Identification of disease target genes

In order to construct the compound-target network, it is important to identify the genes related to disease. The information related to breast cancer associated target genes was collected from GeneCards^32^ (https://www.genecards.org), human gene database which provides information related to all annotated and predicted human genes.

### Construction of compound-target network

Once the protein–protein interaction was carried, the next step is to understand the molecular mechanism which is achieved by constructing the compound-target network using Cytoscape^33^ visualization software v_3.7.1. The compound-target network helps to understand and analyse the mechanism of the components with target as well as the pathway involved.

### Gene Ontology (GO) gene enrichment analysis and Kyoto encyclopaedia of genes and genomes (KEGG) pathway annotation

GO and KEGG pathway annotation is carried on ClueGO, another Cytoscape plug-in which gives a network-based visualization to diminish redundancy of results from pathway enrichment analysis^34^. GO is carried out to analyse the gene cluster in the network to improvise the data prediction. GO provides a hierarchically organized set of thousands of standardized terms for biological processes, molecular functions and cellular components, with curated and predicted gene annotations based on these terms for multiple species. Biological process GO annotation is frequently used resource for pathway enrichment analysis. The study objective is to identify the biological process in order to layout the meaningful functional information. KEGG is used to study gene functions and the metabolic pathway of the inputted network of genes and molecules. It also helps us to find out the contributing pathway of the target associated with the disease.

### Molecular modelling: docking of the active components

Docking study was carried out to find the affinity as well as orientation of the selected active component by docking them against the selected receptors as c-MET, EGFR, PDGFR-α/β, VEGF, and ERR using Glide v_7.6 program interfaced with Maestro v_11.3 of Schrödinger 2017 (Schrodinger, LLC, New York, NY, USA). The crystal structure for c-MET (PDB Id: 5EYD), EGFR (PDB Id: 4ZAU), PDGFR-α/β (PDB Id: 5GRN), VEGFR (PDB Id: 2OH4), and ERR (PDB Id: 1ERR-α) were taken from RCSB Protein Data Bank and prepared for docking using ‘protein preparation wizard’. The structures of compounds were built using Maestro build panel and optimized to lower energy conformers using Ligprep v_3.3^35^.

## Experimental validation

### Western blot analysis

The cell lysates from EAFCP (200 µg/ml)^16^ treated MCF-7 cells were obtained and protein concentrations were measured using a Bradford protein assay kit (Bio-Rad, USA). Approximately, 50 μg lysate was resolved on 10% SDS-PAGE and transferred onto the PVDF membrane (Millipore, USA). The membrane was blocked and incubated with respective primary antibodies (ER and EGFR) at 4 °C overnight. Blots were washed, incubated with HRP-conjugated secondary antibodies, developed using chemiluminescent solution (Immobilon Western, Millipore, USA) and scanned by using gel documentation system (Bio-Rad)^36,37^.

### Animals

All procedure involving animal experiments were reviewed and approved (CPCSEA/IAEC/Pharm.Chem.-31/2016-17/129) by the Institutional Animal Ethical Committee (IAEC) of Y. B. Chavan College of Pharmacy, Aurangabad, India. All experiments were performed in accordance with the regulations and guidelines issued by the Committee for the Purpose of Control and Supervision of Experiments on Animals (CPCSEA) India. Virgin female Sprague–Dawley rats (B.W. 150–210 g) were obtained from Wockhardt Research Centre Pvt. Ltd, Aurangabad, Maharashtra, India, and maintained in standard environment conditions (12:12 h light–dark cycle, 25 ± 2 °C; 55 ± 5% relative humidity) with free access to food pellets and water ad libitum. After adaptation for 7 days, rats were randomly divided into 5 groups of six rats each. On the 50th day of rat’s age, MNU (50 mg/kg) was injected intraperitoneal (i.p.) (Supplementary file S1). A fresh solution of MNU was prepared by dissolving immediately before use in 0.9% NaCl adjusted to pH 4 with acetic acid.

The normal control group I and MNU cancer-induced group II receive normal saline solution, MNU cancer-induced group III and group IV receive treatment EAFCP at 250 and 500 mg/kg, p.o. body weight, while cancer-induced Group V receive TAM 2 mg/kg, p.o. body weight) for 30 days^16^.

### Immunohistochemical analysis

IHC analysis was performed on formalin-fixed, paraffin-embedded tissue sections using standard histologic procedures. The primary antibodies for ER-α was incubated at a dilution of 1:50 for one and half hour at room temperature or 16 h (overnight) at 4 °C. The antigen retrieval was performed with thermic treatment by microwave using 3 cycles of 5 min for ER-α with citrate buffer solution. The tissues were counterstained with hematoxylin. The immunoexpression of ER-α was evaluated as per the Allred score for ER nuclear positivity, the proportion score (PS) (0–5) and the % positive tumor cells are respectively, 0 (0%), 1 (< 1%), 2 (1–10%), 3 (11–33%), 4 (34–66%), and 5 (67–100%). The intensity of staining (IS) for the nuclear positivity of the cells graded as 0, 1, 2, and 3 was as none, mild, moderate, and strong, respectively. So the total scores for ER is given as TS = PS + IS. TS 0 and 2 are negative scores, and 3, 4, 5, 6, 7, and 8 are positive scores^37,38^.

## Supplementary information

Supplementary information
