# Supplementary material for: A network pharmacology-based approach to explore potential targets of Caesalpinia pulcherima: an updated prototype in drug discovery
Source: Sci Rep. 2020 Oct 14;10:17217. doi: 10.1038/s41598-020-74251-1 (PMC7560621; doi:10.1038/s41598-020-74251-1)

**A Network Pharmacology- based Approach to explore potential targets of *Caesalpinia pulcherima*: An updated prototype in drug discovery**

Nikhil S. Sakle, Shweta A. More, Santosh N. Mokale*

Dr. Rafiq Zakaria Campus, Y. B. Chavan College of Pharmacy, Aurangabad-431001, Maharashtra, India

Corresponding author: santoshmokale@rediffmail.com


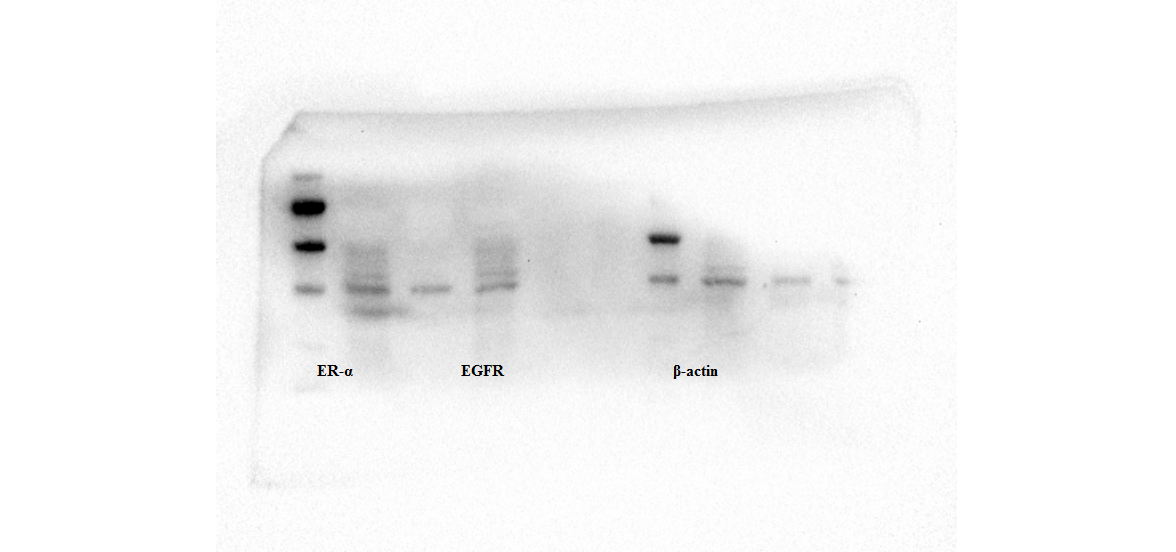

Supplement: Supplementary file 1 — Supplementary information [file 41598_2020_74251_MOESM1_ESM.docx]
